# Addressing the Unmet Hearing Care Needs of Persons Living with Cognitive Impairment: A Qualitative Study of End‐Users, Care Partners, and Experts

**DOI:** 10.1002/alz.086735

**Published:** 2025-01-03

**Authors:** Carrie L Nieman, Marcela D Blinka, Peter Hope, Paul Lee, Xueting Tu, Natalie Wang, Sevil Yasar, Esther S Oh

**Affiliations:** ^1^ Cochlear Center for Hearing & Public Health, Johns Hopkins Bloomberg School of Public Health, Baltimore, MD USA; ^2^ Johns Hopkins University School of Medicine, Baltimore, MD USA

## Abstract

**Background:**

Hearing loss is highly prevalent and can have significant consequences for older adults aging with cognitive impairment. However, few older adults use hearing aids and disparities in care exist by race, ethnicity, and socioeconomic position.

To understand the intersection of hearing loss and cognitive impairment with the ultimate goal of developing an affordable, accessible hearing care intervention responsive to the needs of end‐users, a series of semi‐structured interviews was conducted.

**Method:**

We conducted 20 semi‐structured interviews with 10 participants with mild cognitive impairment (end‐users) and their 10 care partners and 9 experts. End‐users were recruited from the Johns Hopkins Memory and Alzheimer’s Treatment Center. Experts were identified professionals with expertise in dementia, hearing, neuropsychiatric symptoms, healthcare delivery, intervention development, and program implementation and dissemination. We used a heterogeneous purposive sampling to recruit participants from diverse race/ethnicities, education, and income levels. Qualitative content analysis was used to identify themes related to the impact of hearing loss on work, relationships, family life, and neuropsychiatric symptoms along with considerations for the development of a hearing care intervention.

**Result:**

Among end‐users and care partners, 30% self‐identified as African American, 45% as male, and 40% reported less than a college degree (Table 1). End‐users and care partners identified barriers to accessing hearing care, including the stigma associated with hearing loss, the cost of hearing aids, the lack of availability of hearing care, and the participants’ denial of hearing difficulties (Table 2). Suggested facilitators for improving access to hearing care include more affordable hearing aids and incorporating hearing screenings into routine medical care. The participants’ desire to improve communication and care partner support motivate accessing hearing care. Measures of hearing intervention success include strengthened social connections, reduced tension with care partners, and increased social activity. Experts emphasize the importance of education on hearing and dementia, the role of care partners, minimizing burden, and reliance on practical, affordable technology.

**Conclusion:**

In a diverse sample of persons living with cognitive impairment, their care partners, and experts, participants identified unique barriers, facilitators, motivators, and measures of success to guide the development of an affordable, accessible hearing care intervention.

 [Table alz086735-tbl-0001], [Table alz086735-tbl-0002]

**TABLE 1 alz086735-tbl-0001:** Participant Demographics by Participant Type.

	End‐Users: Participants with Mild Cognitive Impairment (*N* = 10)	Care Partners (*N* = 10)	Overall (*N* = 20)
**Demographics**			
Age			
Mean (SD)	79.1 (5.6)	73.8 (7.6)	76.4 (7)
Median [IQR]	80.5 [77.2, 83.5]	75.5 [72.5, 77.5]	77.5 [73.8, 81.2]
Sex			
Male	7 (70.0%)	2 (20.0%)	9 (45.0%)
Female	3 (30.0%)	8 (80.0%)	11 (55.0%)
Self‐ldentified Race/Ethnicity			
African American	3 (30.0%)	3 (30.0%)	6 (30.0%)
White Non Hispanic	6 (60.0%)	7 (70.0%)	13 (65.0%)
Asian	1 (10.0%)	0 (0%)	1 (5.0%)
Education			
Less than college degree	4 (40.0%)	4 (40.0%)	8 (40.0%)
College degree	5 (50.0%)	6 (60.0%)	11 (55.0%)
Skipped	1 (10.0%)	0 (0%)	1 (5.0%)
**Hearing health**			
Better ear pure tone average (dB HL)			
Mean (SD)	38.6 (12.5)	30.9 (9.9)	34.8 (11.7)
Median [lQR]	35 [30.3, 40.6]	28.1 (26.6, 35]	31.2 [27.5, 39.4]
Hearing Status by Categories			
Normal	0 (0%)	2 (20.0%)	2 (10.0%)
Mild	7 (70.0%)	6 (60.0%)	13 (65.0%)
Moderate to severe	3 (30.0%)	2 (20.0%)	5 (25.0%)
Current Hearing Aid Use			
No Current HA use	4 (40.0%)	6 (60.0%)	10 (50.0%)
Prior HA use	6 (60.0%)	4 (40.0%)	10 (50.0%)
***Communication function***			
HHIE‐S			
Mean (SD)	15.4 (12.9)	3.3 (5.2)	9.8 (11.6)
Median [IQR]	20 [3, 27]	0 [0, 7.5]	4 [0, 20]
Missing	3 (30.0%)	4 (40.0%)	7 (35.0%)
**Technology use**			
Email use			
Most days	3 (33.3%)	9 (90.0%)	12 (63.2%)
Some days	1 (11.1%)	0 (0%)	1 (5.3%)
Rarely	1 (11.1%)	1 (10.0%)	2 (10.5%)
None in past month	4 (44.4%)	0 (0%)	4 (21.1%)
Missing	1 (10.0%)	0 (0%)	1 (5.0%)
Computer Use			
Not have/use a computer	2 (22.2%)	1 (10.0%)	3 (15.8%)
Have/use a computer but do not use the internet.	2 (22.2%)	0 (0%)	2 (10.5%)
Have/use a computer and use the internet.	5 (55.6%)	9 (90.0%)	14 (73.7%)
Missing	1 (10.0%)	0 (0%)	1 (5.0%)
Tablet Use			
Not/have a tablet	3 (33.3%)	2 (20.0%)	5 (26.3%)
Have/use a tablet but do not use the internet.	2 (22.2%)	4 (10.0%)	3 (15.8%)
Have/use a tablet or Smartphone and use the internet.	4 (44.4%)	7 (70.0%)	11 (57.9%)
Missing	1 (10.0%)	0 (0%)	1 (5.0%)

**TABLE 2 alz086735-tbl-0002:** Representative quotes from end‐users, care partners, and expert interviews by themes.

	End‐Users and Care Partners
**Barriers to accessing hearing care**	
*Stigma*	I think vanity is a tremendous one. It was for my husband. That's why he got those little teeny things that you couldn't see. Yeah, I think a lot of people don't want to get them because of you can see them. ID 3
*Cost of hearing aids*	As you age, of course, being on a fixed income, you have to make a priority. Do you pay bills? Do you get groceries? Okay, or something of this nature. Well, hearing loss, I'm not too worried about that and everything. It's more important that I be able to buy groceries and put gas in the car. I think it's the cost that really prohibits people from taking positive action. ID 9
*Lack of availability of hearing care*	That's hard because finding any kind of doctor is hard, I mean to know who's really gonna be a good doctor. ID 4
*Participants denial of hearing difficulties*	Like I said, I don't think I have hearing loss. ID 1
**Facilitators**	
*Affordable hearing aids*	… to have an affordable option to correct the hearing deficit with hearing aids. ID 3
*Incorporating hearing screenings into routine medical care*	It probably would help if they could get the hearing test done when they would go to their primary care physicians ID 20
**Motivators**	
*Participants desire to improve communication*	I think it's a—and their relationships. I think if they're married or not married but have a close relationship with a mate, that's a big pusher to get it done. The other is I don't enjoy going to the theater and not being able to hear what's going on on the stage. ID 5
*Care partner supports and motivate accessing hearing care*	I think what makes it easier is, again, the person that they're with and maybe to set up the appointments. ID 6
**Measures of hearing intervention success**	
*Strengthened social connections*	I guess, to me, it seems like the social component, that when people lose that as they get older, it really affects their mood and their ability to contribute their thoughts and their feelings in a way that's not stressful to them, but just that they can be part of the group and benefit from hearing what other people think or say. ID 4
*Reduced tension with care partners*	Well, I think an important example would be if he did pay more attention, and if he was accepting that fact that he may not be hearing well and getting a hearing aid … 1D 8
*Increased social activity*	I guess, to me, it seems like the social component, that when people lose that as they get older, it really affects their mood and their ability to contribute their thoughts and their feelings in a way that's not stressful to them, but just that they can be part of the group and benefit from hearing what other people think or say. ID 4

	Experts
*Education on hearing and dementia*	Dementia education is critical. It has to be. You will be delayed in delivering anything else. I think something about dementia, what we know about the relationship of hearing loss to dementia, it's not caused by dementia, but it can accelerate cognitive loss, and how important attending to hearing is. ID 101
*Role of care partner*	The bigger thing, though, is that you're working with caregivers on making sure that they understand the basics of the device—cleaning, fitting, checking tt. It's definitely more onerous on them. ID 104
*Minimizing burden*	I think the concept—and this is audiology's—one of their biggest problems is that, yes, they want to just give you, here's the prescription, go, let me do my diagnostics and get out. Instead, it's more about how do you integrate it into someone's life. Yeah, 100 percent. ID 104
*Reliance on practical, affordable technology*	What I found interesting is that they could really use their phone a lot. They could use it to find the maps. They could use it to call people, of course. What frustrated them the most are the upgrades, and any changes to apps that meant new learning. That always stuck in mind because they said their phone was their best friend, and they could do so much with it. Then it would be upgraded, and then they can't find where they put their notes, and where—you know what I'm saying? Consistency, is really important, but introducing an approach early on, I think, could be very helpful. ID 101

